# Price transparency at the point of prescribing with real-time prescription benefits

**DOI:** 10.1093/ajhp/zxae108

**Published:** 2024-05-07

**Authors:** Austin Fitts, Andrew J Teare, Scott D Nelson

**Affiliations:** HealthIT, Vanderbilt University Medical Center, Nashville, TN, USA; HealthIT, Vanderbilt University Medical Center, Nashville, TN, USA; Department of Biomedical Informatics, Vanderbilt University Medical Center, Nashville, TN, USA

**Keywords:** clinical decision support, electronic health record, medication adherence, pharmacy benefits, prescribing, real-time prescription benefits

## Abstract

**Purpose:**

Real-time prescription benefits (RTPB) shows prescribers patient-, medication-, and pharmacy-specific information on medication pricing, prior authorization requirements, and lower-cost alternatives. RTPB is intended to improve patient satisfaction and prescription fill rates by decreasing out-of-pocket costs for prescriptions. Therefore, we evaluated how RTPB affects prescribing patterns by examining acceptance and subsequent fill rates for RTPB alternative suggestions.

**Methods:**

RTPB was implemented in February 2022 using external vendor interfaces. Prescribing data from March 2022 to March 2023 were analyzed. RTPB displayed alerts for medications requiring prior authorization or when alternative medications would result in cost savings. Patients were included if their prescription received an RTPB response and they had a subsequent encounter with pharmacy fill data. Primary outcomes were alert acceptance rates and prescription fill rates across RTPB alert groups, with a secondary outcome of monthly copay savings for accepted alerts.

**Results:**

RTPB requests received a response for 88% of prescriptions, with price estimates provided for 77.9% of them. Lower-cost alternatives accounted for 67.2% of alerts, while prior authorization requirements represented 15% of alerts. Prescribers selected a lower-cost alternative 32% of the time. For those with an RTPB alert, patients filled prescriptions 68% of the time when an alternative was chosen, compared to 59% of the time when the original prescription was retained (odds ratio, 1.5; 95% confidence interval, 1.5-1.6; *P* < 0.001). Patients saved an average of $27.77 per month on copay costs when alternatives were selected.

**Conclusion:**

Implementation of RTPB was found to result in significant improvements in prescription fill rates and decrease patient copay costs, despite low alert acceptance rates.

**Key Points**

- Real-time prescription benefits (RTPB) saves patients around $28 per fill.
- RTPB increases patient fill rates when alternatives are accepted.
- Providers accepted RTPB alternatives about 32% of the time.

Prescribers often seek ways to reduce patient out-of-pocket expenses for prescriptions and increase medication adherence. However, patients frequently encounter high costs and/or lengthy delays due to prior authorization (PA) in obtaining their medications. Patients are not the only people affected by these issues; one report estimated that providers spend around 5 hours each week on PAs, pricing consultations, pharmacy call backs to change prescriptions, and searches for lower-cost alternatives to save their patients time and money.^1^

Given the challenges of cost and time delays, patients may not fill their prescriptions, which is known as primary nonadherence. Primary nonadherence poses a significant challenge to effective healthcare delivery and patient outcomes. Studies in the general population have shown that 30% to 50% of patients are nonadherent to their prescribed medication regimens.^2,3^ Nonadherence due to high healthcare and prescription costs occurred in 20% of patients over 65 years old.^4^ A recent meta-analysis looking at primary nonadherence among patients with chronic disease identified higher copay costs and the number of concurrent medications as indicators of higher rates of primary nonadherence.^5^ One pharmacy benefits manager (PBM) found that, when prescribers were shown an alert about PA requirements or lower-cost alternatives, they would commonly switch to a covered alternative.^6^ The same PBM reported that real-time prescription benefits (RTPB), when implemented successfully, was able to save patients $120 to $130 per fill on average, when switching to a lower-cost alternative.^7^

When the prescriber has selected a medication to prescribe, RTPB shows prescribers patient-, medication-, and pharmacy-specific information on how much a medication is estimated to cost, whether there are any PA requirements, and lower-cost therapeutic alternatives. RTPB sends a patient-specific message to the patient’s PBM and returns the message to the provider. The prescriber can then see an accurate estimate for how much the medication will cost (copay) at the selected pharmacy. Once the prescription is signed, RTPB displays an alert to the prescriber if the medication requires PA or if there is an alternative medication that would save the patient money. After viewing the alert, the prescriber can switch the original prescription to a covered alternative, initiate a PA, or continue with the original order.

Recent studies and discussions have highlighted the positive impact of RTPB programs on healthcare outcomes and financial stability.^4,8-10^ However, concerns regarding pricing accuracy and the benefit of implementing RTPB have been raised.^8-10^ The objective of this study was to describe how RTPB affects physician prescribing patterns and primary medication adherence by examining acceptance and subsequent fill rates for alternative suggestions.

## Methods

At Vanderbilt University Medical Center, RTPB was implemented into our electronic health record (EHR) system (Epic Corporation, Verona, WI) in February 2022 using external vendor interfaces with CenterX (AmerisourceBergen, Madison, WI) and Surescripts (Surescripts, Arlington, VA). Prescribing data from March 2022 to March 2023 were analyzed. Pharmacy benefits are verified in the EHR by intake staff using a Surescripts eligibility query at the start of an encounter. After a prescription is entered in the EHR, an RTPB query can be manually requested and routed through the appropriate vendor interface, based on the patient’s pharmacy benefits coverage, and a price for the prescribed medication and potential alternatives are returned to the EHR. Alternative medications include medications within the same class and generic formulations included in the patient’s formulary. The prescriber can then view the price estimate before signing; otherwise, upon their clicking sign, an alert is shown to the prescriber if the medication requires PA or there is an alternative medication that would result in cost savings of at least $0.20 per day and more than $10 per fill. The alert can show up to 3 potential alternatives, all of which are priced at the originally selected pharmacy. RTPB results were returned for all outpatient prescriptions, including discharge orders; inpatient medications and clinic-administered medications were not included.

Patients were included if their prescription received an RTPB response, regardless of whether it showed an alert to the prescriber. To estimate primary adherence, we used pharmacy fill and claims data added to the EHR on subsequent encounters using Surescripts medication history. Medication history data are requested at each patient encounter for those with pharmacy benefits listed in the EHR (eligibility data); therefore, only patients with pharmacy insurance benefits listed in the EHR and a subsequent encounter after they were prescribed the medication triggering the RTPB query were included in the prescription fill rate analysis. The follow-up appointment had to be more than 3 days in the future (to give the patient time to pick up the prescription), and we looked forward 30 days to determine whether the prescription was filled within the next 30 days.

The primary outcomes were the alert acceptance rates and prescription fill rates across RTPB alert acceptance groups, controlling for patient age, sex, race, ethnicity, private or public insurance, the number of active medications, drug status coverage response, pharmacy type, prescriber type, and encounter type. Patients were grouped based on whether they had public insurance, private insurance, both, or neither. We defined private insurance as commercial insurance not provided by a government entity. Public insurance was any government-funded insurance, such as Medicare or Medicaid. Pharmacies were separated by type into retail pharmacies, mail-order pharmacies, and specialty pharmacies based on how they were listed in the Surescripts directory. Retail pharmacies encompass a large group of pharmacies that provide general medication services for common medications. Mail-order pharmacies mail prescriptions to a patient’s home, sometimes even across state lines. Specialty pharmacies provide medications that are typically too expensive for storage in a retail pharmacy, and the prescriptions filled are therefore typically for more expensive medications with fewer cost-efficient alternatives. We counted the number of active medications and organized them into groups of 5. For the secondary outcome of copay savings, if an alternative was selected, we estimated the cost savings to patients using the patient’s copay estimate returned for the original prescription and the patient’s copay price estimate for the alternative medication selected.

For statistical analysis, we used statsmodels and Python 3.10 to fit logistic regression models. We used regularized elastic net regression models to account for interactions between the coefficients and used week number to account for trends over time. Results from the logistic regression models are reported as odds ratios (ORs) with 95% confidence intervals (CIs). We used the *χ*^2^ test to compare categorical variables.

## Results

A total of 446,064 patients received 2,117,785 prescriptions during the study period. Patient demographics are included in Table 1, with 263,723 (59.1%) patients being female. Responses to RTPB queries were received for 1,867,282 (88.2%) prescriptions, with price estimates provided for 1,454,952 (77.9%) of these. RTPB alternative alerts were shown for 87,925 (4.7%) processed prescriptions. Lower-cost alternatives were included in 59,092 (67.2%) alerts, and PA requirements represented 13,230 (15.0%) alerts. A total of 371,065 (83.2%) patients received at least one RTPB response to their prescriptions. Patients saved an average of $27.77 per month, with a median (interquartile range) of $8.02 ($0-$30), on copay costs when an alternative was selected.

**Table 1.** Patient Demographics

Characteristic^a^	Baseline	Alerts shown	Had claims data
Race			
White	318,721 (71.5)	42,611 (69.8)	193,454 (75.9)
Black	52,221 (11.7)	8,706 (14.3)	31,021 (12.2)
Other/unknown	75,122 (16.8)	9,769 (16.0)	30,443 (11.9)
Hispanic	28,318 (6.3)	5,364 (8.8)	16,646 (6.5)
Insurance			
Private	243,570 (54.6)	24,728 (40.5)	132,588 (52.0)
Public	185,686 (41.6)	34,422 (56.4)	112,814 (44.3)
Both	8,343 (1.9)	948 (1.6)	6,094 (2.4)
None	8,465 (1.9)	988 (1.6)	3,422 (1.3)
Sex			
Male	182,333 (40.9)	25,645 (42.0)	100,863 (39.6)
Female	263,723 (59.1)	35,441 (58.0)	154,053 (60.4)
Unknown	8 (0)	0 (0)	2 (0)
Age, years			
<18	101,327 (22.7)	20,879 (34.2)	48,584 (19.1)
18-55	188,830 (42.3)	18,831 (30.8)	101,608 (39.9)
55-65	57,281 (12.8)	6,599 (10.8)	36,015 (14.1)
65-75	57,354 (12.9)	8,403 (13.8)	39,405 (15.5)
75-85	32,485 (7.3)	5,065 (8.3)	23,138 (9.1)
85+	8,787 (2.0)	1,309 (2.1)	6,168 (2.4)
Active medications			
<5	215,503 (48.3)	23,364 (38.2)	84,170 (33.0)
5-10	105,775 (23.7)	14,264 (23.4)	71,129 (27.9)
10-20	96,045 (21.5)	16,348 (26.8)	74,692 (29.3)
20+	28,741 (6.4)	7,110 (11.6)	24,927 (9.8)

^a^Data shown as No. (%).

### Prescribing patterns

Prescribers selected a lower-cost alternative in response to the RTPB alert for 28,441 (32.4%) alerts. As shown in Table 2, prescribers were more likely to select an alternative in the alert if the patient was documented as being African American (OR, 1.2; 95% CI, 1.1-1.2; *P* < 0.001) or having public insurance (OR, 1.3; 95% CI, 1.3-1.4; *P* < 0.001). Drug coverage also had an impact on acceptance, with providers more frequently selecting an alternative when the original medication was covered by insurance with restrictions (OR, 3.0; 95% CI, 2.6-3.3; *P* < 0.001) or was not covered by insurance at all (OR, 10.2; 95% CI, 9.4-11.2; *P* < 0.001). Providers seemed to consider PA status because they were more likely to select an alternative when the original order required PA (OR, 2.5; 95% CI, 2.4-2.8; *P* < 0.001) and less likely to do so when the alternative also required PA (OR, 0.3; 95% CI, 0.3-0.4; *P* < 0.001). Users were less likely to select an alternative if the patient had many other active medications (OR, 0.8; 95% CI, 0.7-0.8; *P* < 0.001) and slightly less likely to do so as time went on (OR, 0.99; 95% CI, 0.99-0.99; *P* < 0.001).

**Table 2.** Covariates Impacting Prescriber Acceptance of RTPB Alerts and Patient Primary Adherence

Variables	Prescriber acceptance of RTPB alert		Patient primary medication adherence	
	OR (95% CI)	*P* value	OR (95% CI)	*P* value
RTPB alert accepted	NA		1.5 (1.5-1.6)	<0.001
Race				
White	Ref.		Ref.	
Black	1.2 (1.1-1.2)	<0.001	0.9 (0.9-1.0)	<0.001
Other	0.8 (0.6-1.1)	0.139	1.1 (0.8-1.6)	0.528
Hispanic	1.0 (1.0-1.1)	0.429	0.9 (0.9-1.0)	0.054
Insurance				
Private	Ref.		Ref.	
Public	1.3 (1.3-1.4)	<0.001	0.6 (0.6-0.6)	<0.001
Sex				
Male	Ref.		Ref.	
Female	1.1 (1.0-1.1)	<0.001	1.0 (1.0-1.0)	0.65
Drug status				
Covered	Ref.		Ref.	
Covered with restrictions	3.0 (2.6-3.3)	<0.001	0.9 (0.8-1.0)	0.04
Not covered	10.2 (9.4-11.2)	<0.001	1.4 (1.3-1.5)	<0.001
Pharmacy type				
Retail	Ref.		Ref.	
Mail order	1.1 (0.8-1.6)	0.678	0.8 (0.6-1.0)	0.049
Specialty	1.1 (0.3-3.2)	0.923	0.5 (0.2-1.1)	0.075
PA status				
PA required on original order	2.5 (2.4-2.8)	<0.001	1.4 (1.3-1.5)	<0.001
PA required on alternative	0.3 (0.3-0.4)	<0.001	0.3 (0.3-0.3)	<0.001
Week number	1.0 (1.0-1.0)	<0.001	1.0 (1.0-1.0)	<0.001
Encounter type				
Office visit	Ref.		Ref.	
Follow-up	1.1 (1.0-1.2)	0.102	0.5 (0.5-0.6)	<0.001
Hospital encounter	1.1 (1.0-1.1)	<0.001	0.5 (0.5-0.5)	<0.001
Orders only	0.9 (0.9-1.0)	0.001	0.9 (0.8-0.9)	<0.001
Telemedicine	0.6 (0.5-0.7)	<0.001	0.9 (0.8-1.0)	0.009
Other	1.5 (1.3-1.6)	<0.001	0.6 (0.6-0.7)	<0.001
Provider type				
Physician	Ref.		Ref.	
Resident/fellow	1.8 (1.7-1.9)	<0.001	0.8 (0.7-0.8)	<0.001
Nurse practitioner	0.7 (0.7-0.7)	<0.001	0.9 (0.9-1.0)	0.006
Physician assistant	0.6 (0.6-0.7)	<0.001	1.3 (1.2-1.5)	<0.001
Midwife	1.8 (1.4-2.2)	<0.001	0.5 (0.4-0.6)	<0.001
Other	0.8 (0.7-0.9)	<0.001	0.9 (0.8-1.0)	0.027
Age, years				
<18	1.4 (1.3-1.4)	<0.001	0.8 (0.8-0.9)	<0.001
18-55	1.4 (1.3-1.5)	<0.001	0.9 (0.8-0.9)	<0.001
55-65	Ref.		Ref.	
65-75	0.9 (0.8-1.0)	0.01	1.2 (1.1-1.3)	<0.001
75-85	0.9 (0.8-1.0)	0.046	1.3 (1.2-1.4)	<0.001
85+	1.0 (0.9-1.2)	0.849	1.6 (1.4-1.8)	<0.001
Active medications				
<5	Ref.		Ref.	
5-10	0.9 (0.8-0.9)	<0.001	0.9 (0.9-1.0)	0.006
10-20	0.8 (0.8-0.9)	<0.001	0.9 (0.9-1.0)	0.054
20+	0.8 (0.7-0.8)	<0.001	0.9 (0.9-1.0)	0.01

Abbreviation: CI, confidence interval; NA, not applicable; OR, odds ratio; PA, prior authorization; ref., reference group; RTPB, real-time prescription benefits.

### Primary adherence

This analysis was limited to patients who had a follow-up encounter in the health system after the medication was prescribed. A total of 254,918 patients met this criterion, with 1,229,069 prescriptions. Of the prescriptions, RTPB returned a price estimate for 953,573 (77.6%) prescriptions, with 62,752 (5.1%) showing an RTPB alert. Of the RTPB alerts, 20,388 (32.5%) were accepted by the prescriber.

The baseline initial fill rate was 61.0% (n = 541,321) for prescriptions that did not display an RTPB alert. Patients filled their prescriptions 68.2% of the time (n = 11,144) when a lower-cost alternative was chosen by their prescriber, compared to 59.4% of the time (n = 19,309) when their prescriber kept the original prescription (Pearson *χ*^2^ test, *P* < 0.001). Patients were less likely to fill their prescription if they were documented as being African American (OR, 0.9; 95% CI, 0.9-1.0; *P* < 0.001), having public insurance (OR, 0.6; 95% CI, 0.6-0.6; *P* < 0.001), or having many other active medications (OR, 0.9; 95% CI, 0.9-1.0; *P* = 0.006). Patients were more likely to fill their prescription if the status of the originally prescribed medication was “not covered” and the user selected a covered alternative (OR, 1.4; 95% CI, 1.3-1.5; *P* < 0.001) or if they were older (OR, 1.6; 95% CI, 1.4-1.8; *P* < 0.001); however, one of the most significant predictors of filling a prescription was whether the prescriber selected an alternative (OR, 1.5; 95% CI, 1.4-1.5; *P* < 0.001).

## Discussion

This study describes the implementation of an RTPB program and its impact on prescribing patterns and primary medication adherence by evaluating RTPB alert acceptance and initial medication fill rates. We found that prescribers selected a lower-cost alternative medication 33% of the time and that patients were 1.5 times more likely to fill their prescription if an alternative was selected.

Initially, prescribers were concerned about the number of potential alerts when RTPB was implemented. Therefore, we first implemented RTPB with very high thresholds for showing an alert (cost savings of at least $2 per day). After review of the data, and following discussion with prescriber groups, we settled on a cost-savings threshold of $0.20 per day in May 2022. Organizations may have different thresholds based on their patient population and prescriber preferences. It is likely that our providers were more accepting of RTPB alerts earlier in the study because the cost savings were perceived to be more significant and became less likely to accept alerts as the price difference decreased later in the study. The threshold will likely need to be modified in the future and when RTPB is implemented in different health systems due to changes in prescribing habits and provider awareness.

Patients in this study were 1.5 times more likely to fill their prescription if their provider accepted an alternative medication suggested by an RTPB alert. Acceptance of an alternative was the most significant predictor of patient fill rates in the logistic regression analysis. Our patient population’s baseline prescription fill rate was around 60%, which is comparable to other reports in large populations, where fill rates ranged from 50% to 70%.^2,3^ Adherence increased to almost 70% after an alternative from an RTPB alert was selected by the prescriber. This increase in prescription fill rate is most likely due to cost savings for patients.

Interestingly, despite the increased rate of acceptance of alternatives in RTPB alerts for patients with public insurance, these patients had lower fill rates, potentially due to low socioeconomic status in the case of Medicaid patients or even the high financial burden of healthcare for Medicare patients, or a combination of these. The increased user acceptance of alternatives for these patients highlights awareness of the financial burden impacting those with Medicaid and Medicare, but this did not directly translate to a substantial increase in prescription fill rates for these patients. Further studies looking directly at the impact of RTPB on Medicare and Medicaid patients should examine the disparities and initiate discussion of opportunities for continued improvement.

The cost savings reported in this study are similar to those in other recently published studies. One study evaluating the direct out-of-pocket cost savings from the implementation of RTPB found an average cost savings of about $28 on a 30-day supply of prescriptions.^9^ This is similar to our average copay cost savings of $27.77 per month when an alternative medication was selected using RTPB. The previous study found alternative medications for 4.2% of prescriptions, which is similar to our alert rate of 4.7%.^9^ Our rate of price estimate retrieval by RTPB was higher than at other centers using similar processes; in another study of 5 medical centers implementing RTPB processes, results were returned for a range of 8% to 60% of prescriptions within the first 3 months of implementation.^10^

### Limitations

There are several limitations to this study and RTPB in general to note. First, not all PBMs participate in RTPB; further, those that participate do not always provide alternatives, and we needed to use multiple vendors to cover the PBMs used in our patient population. There could have been variability in the quality of the alternatives provided by the different PBMs. We also saw that several RTPB queries timed out because it took too long for a response to be sent back to the EHR. This could indicate a need for PBMs to optimize the interconnectivity of data between these platforms, allowing for easier flow of information from one source to another. Another limitation was the availability of pharmacy fill data, which resulted in exclusion of patients without subsequent encounters. This could have led to an overestimate of our effect on fill rates as patients with high rates of follow-up may be more likely to pick up their medication on time and more consistently. Additionally, we did not show alternative information for prescriptions that could be sent to a pharmacy different from the one originally selected, although there may have been additional savings for patients by switching to mail-order pharmacies.

## Conclusion

In conclusion, the implementation of RTPB was demonstrated to lead to significant improvements in prescription fill rates and an average cost savings of $27.77 per month on patient copay costs. The ability to provide real-time prices and alternative medications at the point of prescribing empowers providers to make more informed decisions about their patients’ treatment plans. These findings highlight the potential of RTPB programs as valuable tools to promote patient adherence, medication affordability, and efficient healthcare delivery.

## Data Availability

The data underlying this article cannot be shared publicly due to the privacy of individuals who were included in the study.
